# Erenumab in Chronic Migraine Patients Who Previously Failed Five First-Line Oral Prophylactics and OnabotulinumtoxinA: A Dual-Center Retrospective Observational Study

**DOI:** 10.3389/fneur.2020.00417

**Published:** 2020-05-28

**Authors:** Bianca Raffaelli, Rea Kalantzis, Jasper Mecklenburg, Lucas Hendrik Overeem, Lars Neeb, Astrid Gendolla, Uwe Reuter

**Affiliations:** ^1^Department of Neurology, Charité Universitätsmedizin Berlin, Berlin, Germany; ^2^Clinician Scientist Program, Berlin Institute of Health (BIH), Berlin, Germany; ^3^Praxis Gendolla, Essen, Germany

**Keywords:** chronic migraine, prevention, calcitonin gene-related peptide, erenumab, onabotulinumtoxinA

## Abstract

**Background:** German authorities reimburse migraine prevention with erenumab only in patients who previously did not have therapeutic success with at least five oral prophylactics or have contraindications to such. In this real-world analysis, we assessed treatment response to erenumab in patients with chronic migraine (CM) who failed five oral prophylactics and, in addition, onabotulinumtoxinA (BoNTA).

**Methods:** We analyzed retrospective data of 139 CM patients with at least one injection of erenumab from two German headache centers. Patients previously did not respond sufficiently or had contraindications to β-blockers, flunarizine, topiramate, amitriptyline, valproate, and BoNTA. Primary endpoint of this analysis was the mean change in monthly headache days from the 4-weeks baseline period over the course of a 12-weeks erenumab therapy. Secondary endpoints were changes in monthly migraine days, days with severe headache, days with acute headache medication, and triptan intake in the treatment period.

**Results:** Erenumab (starting dose 70 mg) led to a reduction of −3.7 (95% CI 2.4–5.1) monthly headache days after the first treatment and −4.7 (95% CI 2.9–6.5) after three treatment cycles (*p* < 0.001 for both). All secondary endpoint parameters were reduced over time. Half of patients (51.11%) had a >30% reduction of monthly headache days in weeks 9–12. Only 4.3% of the patients terminated erenumab treatment due to side effects.

**Conclusion:** In this treatment-refractory CM population, erenumab showed efficacy in a real-world setting similar to data from clinical trials. Tolerability was good, and no safety issues emerged. Erenumabis is a treatment option for CM patients who failed all first-line preventives in addition to BoNTA.

## Introduction

Migraine prevention is hampered by poor tolerability of available oral drugs, low therapeutic adherence, and insufficient efficacy in a substantial percentage of patients (1, 2). Prior to the approval of the calcitonin gene-related peptide (CGRP)-(receptor) monoclonal antibodies (mAbs), topiramate, and onabotulinumtoxinA (BoNTA) had been the only approved preventative medications in the United States and Europe for the prophylaxis of chronic migraine (CM) (3). mAbs have shown in clinical trials a favorable profile in terms of safety and efficacy, along with significant improvement in daily functioning and quality of life (4, 5). They have several potential advantages compared to standard oral preventives, including a rapid onset of efficacy, ease of use, persistent therapeutic effect, and lack of pharmacological interactions with other medications (6–8).

Erenumab, which blocks the calcitonin gene-related peptide (CGRP) receptor, was launched in Germany in November 2018 and is approved by the European Medicine Agencies (EMA) for the treatment of CM (9). Approval was based on the phase II registration trial (NCT02066415) (10). In this trial, both erenumab doses (70 and 140 mg) led to a significantly greater reduction of monthly migraine days (MMD) than placebo in the last four of the 12 study weeks (−6.6 days for erenumab vs. −4.2 for placebo) (10). Over two-thirds (73.8%) of the patients in the trial had tried at least one prior preventive treatment, and 92.1% of these reported at least one treatment failure due to poor efficacy or tolerability (11). Of note, 66.5% of patients who had previously tried BoNTA therapy for CM had failed this treatment (11). Failure to respond to more than three preventives previously was an exclusion criterion in this trial (10). Erenumab and other CGRP antibodies have not been studied in a migraine population with more than four treatment failures.

Owing to the lack of evidence that erenumab or any other CGRP mAb is superior to established first-line migraine preventive drugs, the European Headache Federation (EHF) and other international guidelines as well as expert opinion suggest the use of mAbs in patients who failed at least two previous oral prophylactic therapies or BoNTA in CM (6, 12, 13). In Germany, the German Federal Joint Committee (Gemeinsamer Bundesausschuss = GBA), a board that sets medical therapy regulations for the public health insurance sector, has identified a specific group of patients for whom the treatment with a mAb will be reimbursed (14). The suitable group consists of patients who previously failed or had contraindications for at least five different anti-migraine treatment classes. According to the authorities, these include the following first-line preventives: one beta blocker (metoprolol or propranolol), flunarizine, topiramate, amitriptyline, and valproate (14). In CM patients, previous failure to BoNTA is additionally required for the reimbursement of erenumab (14). The rationale for these six recommended classes is not based on rigorous scientific data, but rather on the responsible body's (GBA) majority decision (14). This rule applies to the public health insurance sector, which covers the costs of 90% of the population in Germany. Although not favorable to the patients, the GBA's ruling allows us the real-world analysis of data from a patient population that has, at least to our knowledge, never been studied in a clinical migraine trial.

Therefore, we conducted an analysis of CM patients on erenumab therapy who had previously failed or had contraindications to all first-line oral preventives and additionally BoNTA.

## Materials and Methods

We analyzed the pharmacy prescriptions for erenumab between November 1, 2018 and April 30, 2019 of the headache center at the Charité—Universitätsmedizin Berlin and the headache specialist's practice Praxis Gendolla in Essen, Germany, retrospectively. This was followed by the review of the electronic chart of every patient with a registered erenumab order and the diagnosis of CM. Other headache diagnoses were exclusion criteria. Only patients who received at least one erenumab s.c. injection and also had history of a non-successful BoNTA therapy following the PREEMPT protocol (15) were included in this analysis. In addition, all patients had failed five first-line migraine preventive medications (metoprolol/propranolol, flunarizine, topiramate, amitriptyline, and valproate) or were unsuitable for these therapies due to contraindications.

In line with a recent study (16), failure to previous medications including BoNTA was defined as treatment discontinuation due to lack of efficacy and/or tolerability reasons as self-reported by patient and/or according to physician decision as documented in the patients' chart.

### Headache Characteristics and Clinical Evaluation

We collected headache data for the following periods: 4 weeks before erenumab treatment (baseline), weeks 1–4 after treatment initiation, weeks 5–8 (after the second treatment cycle), and weeks 9–12 (after the third treatment cycle).

Patients recorded their headaches in headache diaries, which are routinely used and collected in our headache centers. The standard headache diary used by our patients is provided by the German Migraine and Headache Society (Deutsche Migräne- und Kopfschmerzgesellschaft, DMKG) and is available in different languages at http://www.dmkg.de/patienten/dmkg-kopfschmerzkalender.html. When headache diaries were not available, we used the electronic documentation of headache data by the treating physician. Headache data in headache diaries or per electronic documentation included the following discrete numerical variables: monthly headache days (MHD), MMD, monthly days with severe headache (MDSH), monthly days with acute medication use (AMD), and monthly days with triptan use (TriD). We collected side effects and dosing information (70 or 140 mg) as categorical variables using the electronic documentation of the treating physician. Only patients with complete information about at least MHD during baseline were included in the efficacy analysis, i.e., analysis of headache characteristics over time. Patients with missing headache data were excluded from the efficacy analysis, but still included in the analysis of side effects.

A headache day was defined as any day on which a patient recorded any type of headache. We classified a headache day as migraine day if the ICHD-3 criteria of probable migraine applied (17), or when headache was preceded by an aura, and/or improved after triptan intake. We defined headache intensity ≥7/10 on a numeric analog scale as severe. All headache data were averaged across the respective 4-weeks period (i.e., at baseline, weeks 1–4, weeks 5–8, and weeks 9–12).

We also assessed multiple demographic and anamnestic features of the study population. This included the categorical variables sex (female or male), family history for headaches (positive or negative), and history of aura (positive or negative), the continuous numeric variables age, and age at migraine onset. For all previous prophylactic medications, we collected the numeric variable treatment duration, and time interval prior to erenumab treatment, and the categorical variable reasons for treatment failure (side effects or lack of efficacy). For BoNTA, we also recorded the number of treatment cycles and documented the side effects in detail.

### Statistical Analysis

Demographic and anamnestic variables were examined using descriptive statistics. The primary endpoint of our analysis was the change in MHD from baseline over the course of a 12-weeks treatment. The secondary endpoints were changes in MMD, MDSH, AMD, and TriD in the same time period. Normal distribution of data was assessed with the Kolmogorov–Smirnov test. Since all variables were normally distributed, we compared the 4-weeks baseline phase with the 4-weeks period following each treatment cycle using paired-samples *t*-tests (i.e., baseline vs. weeks 1–4, baseline vs. weeks 5–8, and baseline vs. weeks 9–12). Patients included in each pairwise comparison varied depending on available headache information. We reported the number of included patients for each analysis. Statistical analysis was performed with IBM SPSS Statistics, version 25. A value of *p* ≤ 0.05 was considered statistically significant. Test for significance was corrected for multiple comparisons using Bonferroni correction. Categorical data were reported as percentage, numerical data as mean (±standard deviation or 95% confidence interval). Owing to the retrospective design of the study, we did not perform a sample size calculation but included all patients fulfilling the inclusion criteria treated at our headache centers between November 1, 2018 and April 30, 2019.

## Results

### Demography

**Figure 1:**
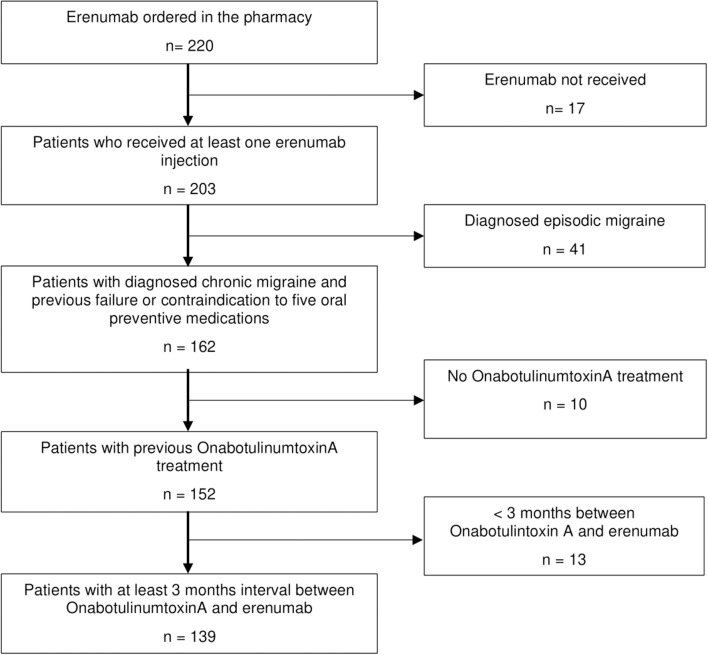
Flowchart of patient selection.

We included 139 CM patients in the analysis (Figure 1). All patients were eligible for erenumab therapy according to the authorities' regulations. Both headache centers contributed patient data in equal numbers [*n* = 71 in Essen (51.1%) vs. *n* = 68 in Berlin (48.9%)].

Patients were mostly female (*n* = 116, 83.5%) with an average age of 53.4 ± 10.2 years; age at migraine onset was 20.0 ± 13.6 years. A history of aura was reported in 31 patients (22.3%), and a large majority (*n* = 115, 82.7%) had a positive family history for migraine. Demographic variables were not different for patients in Berlin and in Essen (Table 1).

**Table 1:** Selected demographic and anamnestic characteristics of patients in our two headache centers.

	**Berlin**	**Essen**	***p***
*N*	71	68	
Female (%)	78.9	88.2	>0.999
Age	52.5 ± 9.7	54.3 ± 10.6	>0.999
Age at migraine onset	19.5 ± 17.0	20.7 ± 9.0	>0.999
History of aura	23.4%	23.9%	>0.999
Family history for headaches	96.2%	76.4%	0.140

*n, number of patients; p, Bonferroni adjusted p-value for multiple (= 5) comparisons*.

### Migraine Prophylactic Treatments

In addition to BoNTA, patients had on average 3.6 ± 1.2 non-successful prior treatment attempts due to lack of either efficacy or tolerability issues. This number does not include medications for which contraindications exist. This was in the majority of cases valproate in women with childbearing potential. The reasons for treatment termination are shown in Table 2.

**Table 2:** Characteristics of previous prophylactic treatment.

**Medication**	***n* (%)**	**Treatment duration (months)**	**End of onabotulinumtoxinA (BoNTA) therapy to erenumab initiation in years**	**Reason for treatment discontinuation**	
				**Side effects**	**Lack of efficacy**
β-Blocker	90.6	29.9 ± 47.9	6.6 ± 5.9	40.3%	95.1%
Topiramate	87.1	20.2 ± 31.1	5.9 ± 4.9	72.4%	81.4%
Flunarizine	65.5	5.2 ± 7.6	6.0 ± 6.3	52.0%	89.8%
Valproate	36.0	3.2 ± 2.9	6.1 ± 6.2	82.6%	91.3%
Amitriptyline	77.4	17.1 ± 26.6	5.2 ± 5.1	61.7%	92.2%

A large majority of patients (*n* = 111, 79.9%) also failed further prophylactic medications of second or third choice (18), most commonly venlafaxine (*n* = 48), candesartan (*n* = 31), or opipramol (*n* = 28).

Twenty patients (14.4%) continued one other concomitant migraine prophylactic treatment (*n* = 7 metoprolol, *n* = 10 topiramate, and *n* = 2 amitriptyline) during erenumab therapy. Three more patients stayed on metoprolol due to arterial hypertension, and seven on amitriptyline because of concomitant depression.

### Historic OnabotulinumtoxinA Treatment

Patients in this analysis had received 4.1 ± 3.8 BoNTA treatment cycles following the PREEMPT protocol (15). Side effects of BoNTA were reported by 17.3% of patients, among which neck pain was the most frequent (37.5%), followed by facial paralysis or ptosis (25.0%), and injection site pain (16.7%). The discontinuation rate due to side effects was 11.5%; all other patients terminated BoNTA due to insufficient headache response. All patients who discontinued BoNTA primarily due to side effects had received either one or two treatment cycles and had not reported a relevant migraine improvement until treatment discontinuation.

### Erenumab Treatment

Between November 2018 and April 2019, *n* = 14 patients had received at least one erenumab treatment cycle: *n* = 26 two, *n* = 32 three, and *n* = 67 more than three treatment cycles in a monthly subcutaneous regimen. Average time interval between the last BoNTA treatment cycle and the first erenumab treatment was 34.8 ± 37.1 months. Patients started erenumab therapy with a dose of 70 mg s.c. without any exception. Dosage escalation to 140 mg was done in 7.3% of patients after 4 weeks (second treatment) and in 29.5% after 8 weeks (third erenumab cycle). A small majority of patients (52.8%) who continued erenumab after the third cycle received thereafter a dose of 140 mg.

### Headache Characteristics During Erenumab Treatment

Eighty-four patients completed headache diaries during the four baseline weeks and reported 18.2 MHD (95% CI 16.8–19.65). MHD at baseline were similar in patients in Berlin (17.7, 95% CI 15.8–19.6) and in Essen (18.9, 95% CI 16.55–21.33, *p* = 0.405). Erenumab led on average to a reduction of MHD by 21.5% (95% CI −30.8−12.1) in weeks 1–4 (*n* = 68), by 31.1% (95% CI −40.1−22.2) in weeks 5–8 (*n* = 60), and by 27.2% (95% CI −37.9−16.4) in weeks 9–12 (*n* = 45, *n* = 25 with 70 mg and *n* = 20 with 140 mg).

Almost 40% of patients (*n* = 27/68) reported a reduction of >30% in weeks 1–4, 53.3% (*n* = 32/60) in weeks 5–8, and 51.1% (*n* = 23/45) in weeks 9–12. A 50% response to erenumab was achieved by one in three patients (31.1%) in weeks 9–12.

**Figure 2:**
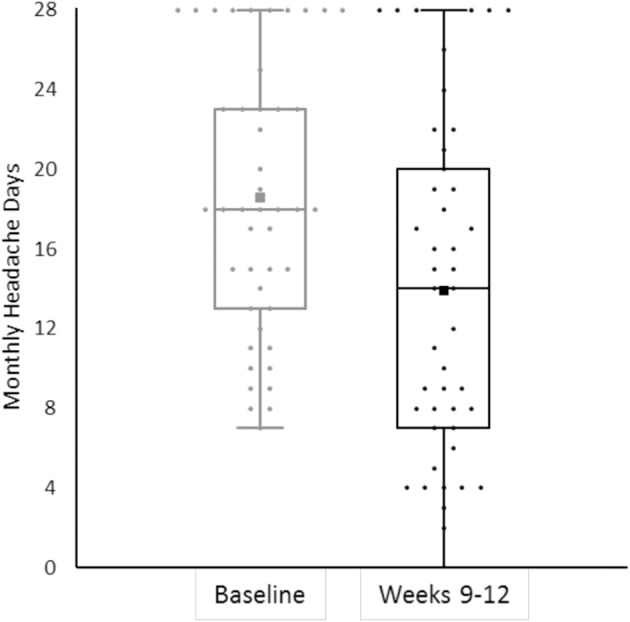
The bee swarm plot shows mean monthly headache days during the 4-week baseline and in erenumab treatment at weeks 9–12 (*n* = 45).

We also had patients without any response to erenumab treatment. Eleven patients (24.4%) showed no change or worsening of MHD in weeks 9–12, in addition to the previous failure to BoNTA and all first-line treatment classes. Figure 2 shows response rates in weeks 9–12.

In a descriptive analysis, patients who continued on erenumab 70 mg seemed to have higher response rates than patients who switched from 70 to 140 mg: −36.6% (95% CI −49.2−24.0) for the 70-mg group and −15.3% (CI −33.8–3.1) for the 140-mg group in weeks 9–12.

Other parameters such as MMD, MDSH, and AMD showed significant improvement (Table 3). In particular, erenumab reduced days with the intake of a triptan by more than 50% during the observation period (baseline 10.7 TriD, 95% CI 9.1–12.3, −4.7 in weeks 9–12, 95% CI 4.6–7.7, *p* < 0.001). Patients with and without another concomitant prophylactic treatment (CCPT) did not differ in the reduction of MHD [−5.4, 95% CI −0.4–11.32 (weeks 9–12) for seven patients with CCPT, −4.5, 95% CI 2.6–6.5 for 38 patients without].

**Table 3:** Headache characteristics during erenumab treatment vs. baseline (4 weeks before erenumab treatment).

	**Weeks 1–4**	**Weeks 5–8**	**Weeks 9–12**
MHD (baseline)	14.0 ± 8.3 (17.7 ± 6.8)	13.4 ± 8.6 (18.7 ± 6.9)	13.9 ± 8.5 (18.6 ± 6.8)
Reduction from baseline	−3.7 ± 5.5	−5.3 ± 5.4	−4.7 ± 5.9
*N*	68	60	45
*P*	<0.001	<0.001	<0.001
MMD (baseline)	10.5 ± 6.4 (14.6 ± 5.3)	10.4 ± 6.7 (15.3 ± 6.0)	10.9 ± 6.4 (15.4 ± 5.0)
Reduction from baseline	−4.0 ± 5.5	−4.9 ± 4.4	−4.5 ± 4.6
*N*	43	38	23
*P*	<0.001	<0.001	<0.001
MDSH (baseline)	3.4 ± 4.3 (6.7 ± 5.8)	3.7 ± 4.7 (7.0 ± 6.3)	3.3 ± 4.3 (7.6 ± 5.4)
Reduction from baseline	−3.3 ± 4.4	−3.3 ± 4.1	−4.3 ± 4.7
*N*	29	23	13
*P*	0.004	0.015	0.09
AMD (Baseline)	7.0 ± 4.4 (11.9 ± 4.6)	7.0 ± 4.3 (12.3 ± 5.7)	6.5 ± 2.9 (12.8 ± 5.0)
Reduction from baseline	−4.9 ± 4.0	−5.3 ± 5.2	−6.3 ± 4.8
*N*	43	35	22
*P*	<0.001	<0.001	<0.001
TriD (Baseline)	6.6 ± 5.7 (10.7 ± 5.9)	5.1 ± 4.0 (10.3 ± 6.9)	5.6 ± 2.8 (10.3 ± 6.2)
Reduction from baseline	−4.1 ± 4.1	−5.2 ± 6.1	−4.7 ± 4.6
*N*	45	39	27
*P*	<0.001	<0.001	<0.001

*MHD, monthly headache days; MMD, monthly migraine days; MDSH. monthly days with severe headache; AMD, monthly days with acute medication use; TriD, monthly days with triptan use; n, patients in the respective category with available data for analysis; p, Bonferroni adjusted p-value for multiple (=15) comparisons. Data are reported as mean ± standard deviation*.

### Tolerability

In total, *n* = 52 (37.4%) patients reported side effects. The most common side effect was constipation (*n* = 26, 18.7%), followed by respiratory tract infections (*n* = 6, 4.3%), and itching at injection site (*n* = 5, 3.6%). Constipation was particularly common in patients with the parallel intake of tricyclic antidepressants: five out of 11 patients (45.1%) in this group reported constipation as a side effect.

The discontinuation rate due to side effects was 4.3% (*n* = 6) during the entire observation period. Patients recorded the following reasons for discontinuation: *n* = 3 worsening of migraine, *n* = 1 skin rash, *n* = 1 new asymptomatic ST depression in ECG, and *n* = 1 constipation.

More than 70% of patients (71.2%, *n* = 99) continued erenumab treatment after April 2019, 21.6% (*n* = 30) discontinued treatment due to insufficient response, and in further 2.9% (*n* = 4) information was missing.

## Discussion

In this retrospective analysis, erenumab showed efficacy in CM patients who failed or had contraindications to five first-line migraine preventives and, in addition, BoNTA. Beginning with the first treatment cycle, erenumab led to a significant reduction in MHD in this difficult-to-treat cohort. Fifty percent of the patients reported a reduction in MHD of at least 30%, which is considered clinically meaningful (19). Migraine frequency reduction led to a reduced number of days with acute medication, in particular triptan, intake. The low discontinuation rate in this analysis indicates good tolerability of erenumab in this population.

This is the first real-world analysis, which assesses erenumab efficacy in CM patients with six prior frustrating treatment attempts (first-line oral medications plus BoNTA). Such patients have not been studied in any phase of the mAb developmental program, but reflect a substantial number of patients in headache centers. Therefore, analyses like ours help to understand the potential of this new medication class in the clinical context of the most refractory patients.

Phases II and III studies for the CGRP and CGRP-receptor mAbs demonstrated efficacy of mAbs in patients who previously did not respond to other preventives. The number of treatment failures was limited to a maximum of two to four in CM trials, with some small differences between trials (11, 16, 20, 21). Findings from our real-world study also show positive results for erenumab in a more refractory patient population.

In the phase II study of erenumab in CM, 34.8% of patients had previously failed three preventive treatments (11). A *post-hoc* analysis in this particular subgroup revealed that 34.8% of the patients with erenumab 70 mg and 38.5% with erenumab 140 mg reached an at least 50% response in MMD vs. 15.3% in the placebo group (11). This is highly consistent with our findings, with over 30% of the patients achieving at least 50% response after 3 months of treatment. The LIBERTY trial focused specifically on patients who had failed two to four preventive treatments (20). Although this trial enrolled only patients with episodic migraine and a direct comparison with our analysis is not possible, responder rates were remarkably similar to our study population: in fact, three out of 10 patients on erenumab treatment reached at least 50% response (20).

The dosing of erenumab is still a matter of discussion (22). In the EM STRIVE trial, but not the CM trial, erenumab patients achieved a larger reduction of MMD with a dose of 140 mg rather than 70 mg at the time of the primary endpoint (10, 23). At the end of the open-label extension in both EM and CM, the 140-mg dose showed a numerically higher reduction of MMD than the 70-mg dose (24). In our headache centers, treatment initiation at the time of the analysis was done in line with the EMA approval of erenumab with 70 mg followed by an increase to 140 mg if the patient did not respond sufficiently. Therefore, it is not surprising that patients who were stable on 70 mg achieved higher response rates than those who switched to 140 mg as this population is more likely to be overall less responsive to erenumab. However, this analysis was purely descriptive. A dedicated outcome study is necessary to confirm this finding.

In randomized double-blind and open-label trials, erenumab demonstrates a good tolerability profile, and also, in our real-world study, only a few patients discontinued treatment due to adverse events. The most common side effect in our cohort was constipation (18.7%), which is considerably higher than in the STRIVE trial, in which about 3.5% of the patients reported constipation (23). Several factors may contribute to higher constipation rates in a real-world setting such as predisposition, co-medication with drugs that have an influence of gut mobility (e.g., antidepressants) or specific patient information before the initiation of erenumab therapy. In line, constipation rates were particularly high in patients with concomitant tricyclic antidepressant therapy, and treatment with erenumab in this patient population should be carefully evaluated.

Real-world experience with erenumab is still limited. Initial reports in an Italian headache center included 65 patients with CM who had received at least one injection of erenumab (25). These patients had 5.4 ± 2.6 prior treatment failures; data on prior medication classes including BoNTA was not reported (25). In this study, eight patients had received at least two treatment cycles of erenumab by the time of publication. MMD decreased by 6.6 ± 4 at week 8 in this population, which corresponded to an outstanding 50% responder rate of 87.5% (25). We did not reproduce these findings in our sample, possibly due to population differences and a longer observation period. A placebo response is typically reduced with a longer treatment duration.

The first data from two Australian headache centers with 64 patients who had failed at least three previous preventive medications showed a >50% reduction in MHD in 30% of cases after 3 months of treatment (26). This is in line with our findings.

In a recently published observational trial of 89 Italian patients with episodic or chronic migraine, 61.8% of the patients reached a 30% response rate after the third treatment cycle with erenumab. In this cohort, only 11 patients (12.4%) had more than four previous treatment failures, which may lead to better response rates to erenumab than in our patient group. However, in a subgroup analysis of CM patients who previously failed BoNTA treatment, 56.8% achieved a 30% response, which is comparable to our results (27). The identification of clinical or laboratory parameters associated with a good treatment response could help us in the selection of patients for successful CGRP mAb therapy in the future (28).

The antinociceptive action of BoNTA is partially mediated by the inhibition of CGRP release from trigeminal nerve fibers (29, 30). Efficacy of erenumab in BoNTA non-responders indicates that the mechanisms of action do not fully overlap. One possible explanation is the abundance of erenumab in the entire circulation, while BoNTA has rather a local effect on CGRP release at the injection site (31).

German treatment guidelines recommend efficacy evaluation of BoNTA after three treatment cycles (32). In our analysis, BoNTA non-responders had received more than four BoNTA treatments on average, which seems in contrast to the guideline recommendations. The following explanations may apply: patients had negative experience with oral preventatives and experienced some improvement related to pain intensity under BoNTA treatment with no or very few side effects. These patients usually stayed on BoNTA treatment until a switch to mAb treatment was possible. In some patients, the placebo response associated with BoNTA injections may have contributed to an initial treatment success. Placebo effects get lower over time. We know from previous literature that a diminished benefit after long-term treatment is possible, even if rare (33). Because the BoNTA treatment period was not the scope of this analysis, we did not collect headache days during this epoch. In the chart review, we detected higher discontinuation rates from BoNTA treatment due to side effects (11.5%) than in the PREEMPT trials (3.8%) or in real-world analyses (15, 33). Because this analysis focused on patients who failed BoNTA treatment due to safety or tolerability issues, we may have a bias toward patients with poor tolerability.

The main limitations of our study are the retrospective character and missing data points. Patients are requested to complete headache diaries before treatment initiation and during treatment with mAbs as part of our clinical routine. However, a number of patients fail to provide their calendars regularly, and therefore, data is lacking. As a consequence, analyses were limited to a comparison of individual time point vs. baseline using *t*-tests rather than analysis of variance over all timepoints. Owing to better data quality for headache days rather than migraine days only, we considered MHD as a primary endpoint and calculated response rates on the basis of MHD. Based on our clinical experience, in this cohort of patients with CM and without any other headache disorder, headache days mostly correspond to migraine days, and the decrease in MHD closely resembles the decrease in MMD. Analysis of response included only patients with complete data for MHD. Patients with missing data for any reasons, including previous treatment discontinuation, were excluded. Moreover, patients with a good treatment response may be inclined to fill their headache diary in a more accurate way. This might have caused a selection bias toward overrepresentation of patients with higher response rates.

In conclusion, this real-world analysis of erenumab complements clinical trial results and suggests that erenumab shows good efficacy and tolerability even in patients who failed all first-line prophylactic treatments plus BoNTA. Our analysis indicates efficacy of erenumab in a patient population for which no data from randomized placebo controlled trials exist.

## Data Availability Statement

The datasets generated for this study are available on request to the corresponding author.

## Ethics Statement

Ethical review and approval was not required for the study on human participants in accordance with the local legislation and institutional requirements. Written informed consent for participation was not required for this study in accordance with the national legislation and the institutional requirements.

## Author Contributions

BR and UR: study concept and design, drafting of the manuscript. BR, RK, AG, and UR: acquisition and collection of data, data analysis, and interpretation. JM, LO, LN, and AG: critical revision of the manuscript for important intellectual content. All authors: approval of the final version of the manuscript.

## Conflict of Interest

BR has received research funding and honoraria from Novartis Pharma, TEVA, Hormosan, and Pharm Allergan. RK declares no conflict of interest. JM has received honoraria from Novartis Pharma. LO declares no conflict of interest. LN has received honoraria from Novartis Pharma, Eli Lilly, Pharm Allergan, Desitin, Hormosan, and TEVA. AG has received honoraria from Novartis Pharma, Pharm Allergan, Desitin, Autonomic Technologies, Medtronic, Grnenthal, Mundipharma, MSD, TEVA, Hormosan, und Reckitt Benckiser. UR has received honoraria from Novartis Pharma, Amgen, Pharm Allergan, Autonomic Technologies, Co-Lucid, Eli Lilly, Medscape, StreaMedUp, and TEVA. BR, JM, LN, AG, and UR are also involved as investigators in clinical trials with monoclonal antibodies from Amgen, Alder, Eli Lilly, Novartis, and TEVA without personal remuneration.
